# Monitoring surface dynamics of electrodes during electrocatalysis using *in situ* synchrotron FTIR spectroscopy

**DOI:** 10.1107/S1600577523000796

**Published:** 2023-02-16

**Authors:** Weiren Cheng, Yanzhi Xu, Chenyu Yang, Hui Su, Qinghua Liu

**Affiliations:** a National Synchrotron Radiation Laboratory, University of Science and Technology of China, Hefei, Anhui 230029, People’s Republic of China; b Institute for Catalysis, Hokkaido University, Sapporo 001-0021, Japan; RIKEN SPring-8 Center, Japan

**Keywords:** *in situ* cell, FTIR spectroscopy, synchrotron radiation, surface dynamics, electrocatalysis

## Abstract

Monitoring the surface dynamics of catalysts under working conditions is important for a deep understanding of the underlying electrochemical mechanisms towards efficient energy conversion and storage. Fourier transform infrared (FTIR) spectroscopy with high surface sensitivity has been considered as a powerful tool for detecting surface adsorbates, but it faces a great challenge when being adopted in surface dynamics investigations during electrocatalysis due to the complication and influence of aqueous environments. This work reports a well designed FTIR cell with tunable micrometre-scale water film over the surface of working electrodes and dual electrolyte/gas channels for *in situ* synchrotron FTIR tests. By coupling with a facile single-reflection infrared mode, a general *in situ* synchrotron radiation FTIR (SR-FTIR) spectroscopic method is developed for tracking the surface dynamics of catalysts during the electrocatalytic process. As an example, *in situ* formed key *OOH is clearly observed on the surface of commercial benchmark IrO_2_ catalysts during the electrochemical oxygen evolution process based on the developed *in situ* SR-FTIR spectroscopic method, which demonstrates its universality and feasibility in surface dynamics studies of electrocatalysts under working conditions.

## Abstract 2

A novel bifunctional *in situ* Fourier transform infrared (FTIR) cell has been elaborately designed and prepared. By coupling with single-reflection infrared mode, a facile and general *in situ* synchrotron FTIR spectroscopic method has been developed based on the FTIR cell for surface dynamic studies of electrodes during electrolysis.

## 1. Introduction

High-performance electrochemical devices, such as electrolyzers, fuel cells, Zn–air batteries *etc.*, are very important for the blossoming of a modern sustainable energy industry (Zhou *et al.*, 2022 ▸; Cheng *et al.*, 2019 ▸; Pomerantseva *et al.*, 2019 ▸). To develop efficient electrochemical devices, one of the key challenges is to design advanced catalysts with high activity and durability to overcome the substantial thermodynamic reaction barriers on their anodes/cathodes (Pomerantseva *et al.*, 2019 ▸; Cheng *et al.*, 2021 ▸). Monitoring the dynamic evolution of electrodes, especially surface species, under working conditions in these electrochemical devices will be very helpful for understanding their intrinsic energy conversion mechanisms for design, synthesis and screening of advanced electrocatalysts (Miyanaga, 2021 ▸; Su *et al.*, 2020 ▸; Cheng *et al.*, 2019 ▸). However, it is still very challenging to promptly probe the progress of reactive intermediates over the electrode surface during the electrocatalytic process due to their low concentration and the substantial influence of aqueous electrolytes (Li *et al.*, 2020 ▸; Catlow *et al.*, 2020 ▸). Hence, a cutting-edge measuring technique with an ultra-high surface sensitivity is urgently needed for surface dynamics studies during electrocatalysis. Fourier transform infrared (FTIR) spectroscopy, which is highly sensitive to surface adsorbed species, has been widely adopted to detect reactants or products on the surface of nanomaterials in various catalytic reactions (Catlow *et al.*, 2020 ▸; Li *et al.*, 2020 ▸; Marcelli *et al.*, 2012 ▸). Moreover, with molecular fingerprint identification of surface active intermediates and relatively facile operation, *in situ* FTIR spectroscopy has been considered a reliable and powerful tool for tracking the surface species evolution of electrodes during electrocatalysis in relation to Raman spectroscopy and X-ray photoelectron spectroscopy (Catlow *et al.*, 2020 ▸; Han *et al.*, 2021 ▸; Huang *et al.*, 2020 ▸).

For *in situ* FTIR measurements during electrolysis, it is a rather challenging task to acquire reliable and high-quality FTIR data due to the dramatic infrared (IR) adsorption of water molecules in aqueous electrolytes (Li *et al.*, 2020 ▸; Nayak *et al.*, 2018 ▸). A well designed *in situ* FTIR cell coupled with a reasonable IR reflection mode will be an effective approach to avert the overwhelmingly weak IR signal of interest in the background IR adsorption of aqueous solutions during *in situ* FTIR tests (Zhang *et al.*, 2014 ▸; Nakamura & Nakato, 2004 ▸; Zhang *et al.*, 2018 ▸). Indeed, a very delicate IR window of ZnSe or diamond single-crystal prisms, which possesses a unique trapezoid section, has been carefully developed for attenuated total reflection infrared (ATR-IR) spectroscopy tests, where the incident IR beam would undergo multiple total reflections within the ZnSe/diamond prisms before being collected by deuterated triglycine sulfate (DTGS) or mercury cadmium telluride (MCT) detectors (Nakamura & Nakato, 2004 ▸; Zhang *et al.*, 2018 ▸; Nayak *et al.*, 2013 ▸). Based on a traditional thermal IR source (Globar), weak IR signals of the targeted surface species can be effectively intensified by approximately one order of magnitude through a multiple reflection mode, which is beneficial for acquiring high-quality FTIR data during *in situ* tests. It is notable that a special optical system is highly imperative for realizing multiple total IR reflections within the ZnSe or diamond single-crystal prisms, which undoubtedly complicates the operation process of the ATR-IR measurements (Zhang *et al.*, 2018 ▸; Li *et al.*, 2020 ▸). Moreover, direct deposition of catalysts onto the IR cell window of ZnSe or diamond prisms as film-like electrodes is required for an effective ATR-IR test, which is difficult and inconvenient to realize for common powdered catalysts. Relative to conventional Globar sources, a synchrotron radiation (SR) IR source shows substantial merits of two to three orders of magnitude greater brightness for SR-based FTIR (SR-FTIR) spectroscopy when the IR aperture size is cut down to a scale of several micrometres (Hu *et al.*, 2020 ▸; Li *et al.*, 2020 ▸; Lee *et al.*, 2020 ▸; Kamel *et al.*, 2021 ▸). Therefore, based on the brighter synchrotron IR source, it is potentially possible to acquire high-quality FTIR spectra under aqueous conditions via a single-reflection mode. For the implementation of effective *in situ* SR-FTIR tests, the design of a smart *in situ* FTIR cell that is compatible with SR IR beamline configurations and the single-reflection mode is absolutely imperative (Diaz-Lopez *et al.*, 2020 ▸; Li *et al.*, 2020 ▸), but has been rarely reported.

Herein, a novel bifunctional *in situ* FTIR cell has been elaborately designed and developed for practical *in situ* SR-FTIR measurements. It is compatible with a single-reflection synchrotron FTIR mode concurrently developed by our group and successfully commissioned at the IR beamline BL01B of National Synchrotron Radiation Laboratory (NSRL), China. Thanks to a unique screw assembly of the IR window, the water film over the surface of working electrodes can be facilely and rationally tuned on a micrometre scale in the well designed *in situ* FTIR cell. Moreover, dual electrolyte/gas channels within the *in situ* FTIR cell enable it to act as a bifunctional *in situ* cell for tracking the surface dynamics of catalysts in various electrochemical reactions of interest that may or may not involve gaseous reactants. Taking commercial benchmark IrO_2_ catalysts as an example, we promptly track the reactive intermediate evolution over the surface of IrO_2_ catalysts during the intriguing electrochemical oxygen evolution reaction (OER) using the well designed *in situ* FTIR cell coupled with the developed single-reflection SR-FTIR method. Based on the *in situ* FTIR observations, it is revealed that the formation of a key *OOH intermediate during the OER may be promoted by the newly formed surface Ir—O bonds during the OER, resulting in fast OER kinetics for commercial IrO_2_ catalysts in alkaline solution. This result demonstrates the feasibility of the developed *in situ* synchrotron FTIR spectroscopic method in dynamic studies of advanced catalysts during electrocatalysis. Compared with traditional FTIR spectroscopy, SR-FTIR with single-reflection IR mode is a more promising approach to acquire reliable and effective IR signals of key intermediates over an electrode surface during electrochemical reactions in a relatively easy operation manner.

## 2. *In situ* FTIR cell

### 2.1. Schematic design

**Figure 1:**
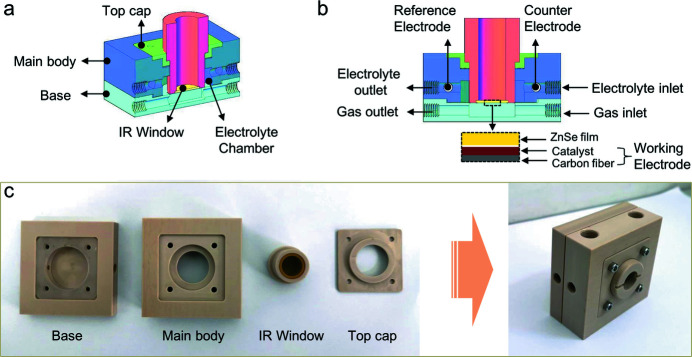
Schematics of the *in situ* FTIR cell observed from the side (*a*) and from the front (*b*). (*c*) Photographs of the *in situ* FTIR cell parts (left) and fully assembled (right).

As shown in Figs. 1 ▸(*a*) and 1(*b*), the well designed *in situ* FTIR cell consists of four main parts: base, main body, top cap and IR window. All these components are processed using polyether ether ketone (PEEK) as the original material, which possesses robust acid–base resistance (pH = 1–14) and can work normally under a wide temperature range of −100°C to 260°C (Ma *et al.*, 2020 ▸; Ling *et al.*, 2020 ▸). For the IR window, a round ZnSe film, of diameter ∼14 mm and thickness ∼1 mm, is fixed at the end of a hollow cylindrical PEEK tube; IR radiation can pass through the tube and ZnSe film from the inside to easily arrive at the working electrode. The working electrode is prepared by homogeneously coating catalysts onto a square hydro­phobic conductive carbon cloth, which will be placed as close as possible to the ZnSe film of the IR window [Fig. 1 ▸(*b*)]. Note that the IR window is attached to the main body via a thread assembly, where a special thread spacing of approximately 0.2 mm is adopted; a thread feed of about 5 µm is expected to move the IR window forward/backward to/from the working electrode with every 10° rotation of the cylindrical IR window. This is very helpful to precisely adjust the thickness of the electrolyte layer among the ZnSe film of the IR window and the working electrode in the *in situ* FTIR cell.

As presented in Fig. 1 ▸(*a*), there is an electrolyte chamber within the main body of the cell with a volume of approximately 3.0 ml around the assembled IR window. Coupled with the electrolyte inlet and outlet, a flow field of electrolytes surrounding the catalyst layer of the working electrode is created in the main body [Fig. 1 ▸(*b*)], which is beneficial for avoiding the mass transport limitation during electrolysis (Li *et al.*, 2020 ▸; Chen *et al.*, 2020 ▸). Moreover, a gas transport channel is situated just below the working electrode [Fig. 1 ▸(*b*)], from which gaseous products during the electrocatalytic process can readily escape. In the meantime, if necessary, gaseous reactants can be pumped into the cells directly through the gas channel and are then easily accessible to the working electrodes during electrocatalytic reactions that involve gaseous reactants; for example, electrochemical O_2_ reduction, CO_2_ reduction and N_2_ fixation reactions. The well designed electrolyte and gas channels enable the *in situ* FTIR cell to act as a bifunctional cell for dynamic studies of various important electrocatalytic processes with or without gaseous reactants. In addition, a reference electrode and a counter electrode are placed in the electrolyte chamber through the designed holes in the main body, which will be coupled with the working electrode to form the desirable three-electrode system for the *in situ* electrochemical measurements.

### 2.2. Entity

The components of the base, main body and top cap can be easily assembled to generate the preliminary *in situ* FTIR cell using four screw fasteners, as shown in Fig. 1 ▸(*c*). It is worth noting that the square working electrode with length size of 40 mm will be positioned between the electrolyte chamber and the gas channel, where the hydro­phobic carbon-cloth substrate can serve as a useful membrane to prevent the aqueous solution escaping from the electrolyte chamber (Nagy *et al.*, 2020 ▸; Xing *et al.*, 2021 ▸). Meanwhile, a translucent rectangular silicone rubber washer is coupled with the carbon-cloth substrate to further seal the electrolyte chamber and avoid leakage of aqueous electrolyte. Copper tape is placed on top of the carbon-cloth substrate and is tightly connected to it through the four fastening screws; this will serve as the current collector bridging the working electrode and the electrochemical workstation. The IR window is directly screwed into the main body via a thread assembly, where the distance between the IR window and working electrode is tunable by adjusting the number of turns and rotation angle. To clearly depict each component, the assembled *in situ* FTIR cell is placed upright, rather than lying down, on a table in the right-hand image of Fig. 1 ▸(*c*). The two holes on the left-hand side of the assembly are for connecting to a peristaltic pump or gas cylinder via small plastic pipes to form the electrolyte circulating or gas pumping systems, respectively, while the holes on the top of the assembly are for Ag/AgCl reference and carbon counter electrodes. Accordingly, a light and handy cubic-like *in situ* FTIR cell of size ∼60.0 mm × 60.0 mm × 29.0 mm (length × width × height) is developed, which is very easy to assemble and transport, for *in situ* FTIR measurements.

## 3. Implementing *in situ* FTIR measurements

### 3.1. Materials

The commercial IrO_2_ catalysts, Nafion 117 (∼5 wt%) and KOH reagent (99.9%) were purchased from Sigma-Aldrich. The carbon and Ag/AgCl electrodes were obtained from Gaossunion Photoelectric Technology Co. Ltd, Tianjin, China. The hydro­phobic carbon cloth (HCP330P) and conductive copper tape were bought from Shanghai Hesen Electrical Co. Ltd, China. The peristaltic pump and small plastic pipes were obtained from Shanghai Kamoer Co. Ltd, China. Nitro­gen (99.9%) was supplied by Nanjing Special Gas Co. Ltd, China. Laboratory-made deionized water (DI) was used in electrode preparation and electrochemical measurements. All chemicals and commercial IrO_2_ catalysts were used directly without further purification or treatments.

### 3.2. Electrochemical measurement

Electrochemical oxygen evolution measurements were carried out in a 1.0 *M* KOH aqueous solution in the *in situ* FTIR cell using a three-electrode electrochemical workstation (CHI760D, CH Instruments), where commercial IrO_2_ catalysts, carbon rod and Ag/AgCl were adopted as the working, counter and reference electrodes, respectively. All the potentials were converted using a reversible hydrogen electrode (RHE) based on the following equation: *E*_RHE_ = *E*_Ag/AgCl_ + (0.059 × pH) + 0.197 V (Garcia & Koper, 2018 ▸; Brewer *et al.*, 2012 ▸). The overpotential during the OER was determined using the following equation: η = *E*_RHE_ − 1.23 V (Cheng *et al.*, 2020 ▸; Garcia & Koper, 2018 ▸). Linear sweep voltammetry (LSV) curves were recorded in the potential range 1.0–1.7 V versus RHE at a scan rate of 10 mV s^−1^. Regarding the preparation of the working electrode, 5 mg IrO_2_ catalysts were dispersed into a mixed solution of 250 µL DI water, 700 µL ethanol and 50 µL Nafion solution via ultrasonication for 30 min. Afterwards, 900 µL of the resultant catalytic ink was loaded onto a piece of carbon cloth (4 cm × 4 cm) with an effective loading area of 3 cm × 3 cm and a loading mass of 0.5 mg cm^−2^. This as-obtained IrO_2_ catalyst supported on carbon cloth served as the working electrode after drying naturally at room temperature for 6 h.

### 3.3. FTIR spectra collection

#### 3.3.1. Measurement setup

An *in situ* single-reflection synchrotron FTIR method has been developed by our group for dynamic studies over the solid–liquid interface of electrodes during electrolysis, based at the infrared beamline BL01B of Hefei Light Source (HLS) at NSRL, China (Su *et al.*, 2020 ▸; Cheng *et al.*, 2019 ▸). After the upgrade of HLS in 2016, this infrared beamline was reconstructed with upgraded station performance, where IR radiation is extracted from a bending magnet with a superior acceptance angle of 65 mrad × 55 mrad (H × V) and excellent flux of approximately 10^13^ photons s^−1^ (0.1% bandwidth)^−1^ (Hu *et al.*, 2020 ▸). Moreover, an advanced Bruker 70v FTIR spectrometer (Bruker Corporation, Ettlingen, Germany) with a KBr beam-splitter and various detectors (a liquid-nitro­gen-cooled MCT detector was used here) are available at this IR endstation. By coupling with an infrared Bruker Hyperion 3000 microscope, it can provide a broad range of ∼650–4000 cm^−1^ and a high spectral resolution of 0.25 cm^−1^ for IR spectroscopy measurements at the micrometre scale.

**Figure 2:**
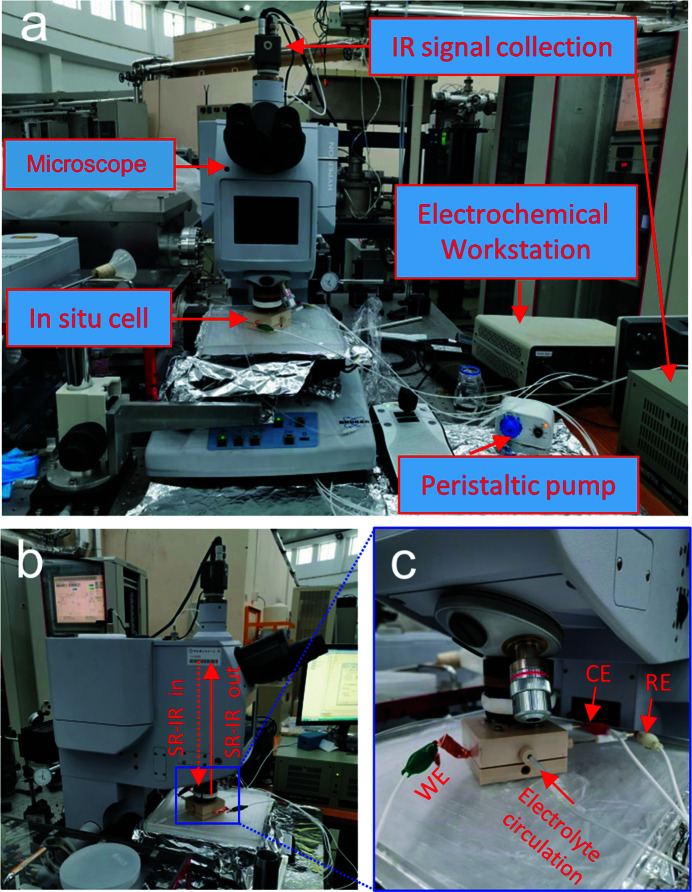
(*a*) Photograph of the equipment used for the *in situ* SR-FTIR measurements. (*b*) Enlarged photograph of the testing microscope instrumentation. (*c*) Enlarged photograph of the *in situ* cell, where WE, CE and RE are the working, counter and reference electrodes, respectively.

As shown in Fig. 2 ▸(*a*), the *in situ* FTIR cell lies on the working platform of the microscope, with the IR window facing up and close to the objective of the microscope. For the electrolyte circulation system, a peristaltic pump is connected to the *in situ* cell using small plastic pipes; 150 mL 1.0 *M* KOH electrolyte contained in a glass bottle forms the external solution reservation. The working electrode is connected to the electrochemical workstation using copper tape, as presented in Figs. 2 ▸(*a*) and 2(*c*), where the counter and reference electrodes are linked to the electrochemical workstation simultaneously. It is noted that the incident IR radiation is almost perpendicular to the surface of the working electrode with a unique incident angle of about 83°, and the reflected IR signals will be collected directly after a single reflection on the surface of the catalysts [Fig. 2 ▸(*b*)]. The IR signals are then recorded and analyzed by the IR signal collection system, and the desirable FTIR spectra are displayed on a computer screen. As illustrated in Fig. 2 ▸(*c*), the IR window of the *in situ* cell is tight to the objective of the microscope, which is useful for shortening the IR light path in air to mitigate the influence of external noise, and is also beneficial for precisely focusing the incident IR beam on the surface of the targeted catalysts.

#### 3.3.2. Measurement procedure

Before *in situ* FTIR measurements were made, highly reflective Au substrate was placed at the catalyst position in the *in situ* cell (Fig. 1 ▸) without electrolyte and the corresponding FTIR spectrum was collected as a reference spectrum in single-reflection mode. Subsequently, FTIR spectra of targeted samples are measured under similar conditions to clarify the intrinsic IR characteristic peaks of the targeted catalysts. During the *in situ* FTIR tests, a FTIR spectrum of the targeted catalyst is firstly collected for the catalyst in electrolyte without applied potentials; this serves as the background spectrum for those acquired under various applied potentials during the electrochemical process. For each selected potential, a constant potential method is adopted, where the potential is applied to the working electrodes in the circulating KOH electrolyte for about 20 min before collection of the FTIR spectrum.

As an example, the surface dynamics of commercial IrO_2_ catalysts during the OER process are promptly monitored using the *in situ* FTIR cell coupled with the developed single-reflection synchrotron FTIR method. The KOH electrolyte in the glass bottle is first bubbled with nitro­gen gas for 30 min to fully purge the dissolved O_2_ in the solution before commencing electrolyte circulation. To acquire high-quality and reliable FTIR data, the working electrode is placed tight to the ZnSe film of the IR window in the *in situ* cell, where there is a water film thickness of about 10 µm over the working electrode surface due to capillary permeation of the porous carbon-cloth substrate. Meanwhile, the incident IR beam is meticulously focused on the targeted IrO_2_ catalysts that are supported on the carbon-cloth, with a micrometre-scale rectangular light spot of 10 µm × 10 µm.

Subsequently, LSV curves were recorded for the IrO_2_ catalysts during the OER to carefully determine suitable applied voltage parameters for the *in situ* FTIR measurements. In general, FTIR spectra are collected at: the open circuit voltage (OCV); the pre-catalytic stage; the onset potential (corresponding to 1 mA cm^−2^); the highly efficient catalytic stage. During the *in situ* FTIR test, a constant potential method is adopted and the electrolyte in the *in situ* cell is kept in close circulation with a flow rate of 50 µL s^−1^. Typically, for the collection of each FTIR spectrum, the selected constant potential is applied to the working electrodes in the circulating KOH electrolyte for 20 min as a pre-treatment step. After that, the FTIR spectra are collected for the IrO_2_ catalysts in the range 650–4000 cm^−1^ with a step interval of 2.0 cm^−1^ by averaging 256 scans. Moreover, the acquired FTIR data are further processed using *OPUS* software (Bruker Optik GmbH, Ettlingen, Germany) to obtain a series of desirable transmission FTIR spectra (Ji *et al.*, 2020 ▸; Kong & Liu, 2021 ▸).

## 4. Results

**Figure 3:**
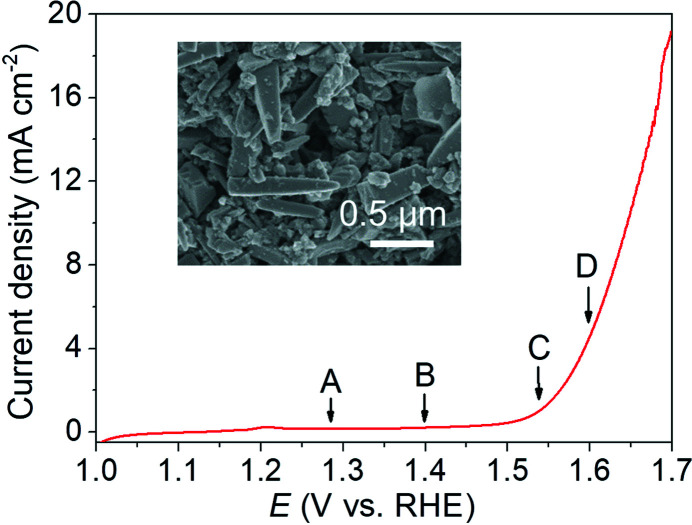
LSV curve recorded using the *in situ* cell without IR correction. The insert shows a transmission electron microscope image of the commercial IrO_2_ catalysts.

The LSV curve in Fig. 3 ▸ shows that the commercial rod-like IrO_2_ catalyst has high OER activity in alkaline solution with an onset potential of 1.55 V versus RHE (at 1 mA cm^−2^) and a good overpotential (η) of about 0.41 V at 5 mA cm^−2^ (corresponding to a potential of 1.64 V versus RHE), which is consistent with previous reports (Plevová *et al.*, 2021 ▸; Zheng *et al.*, 2022 ▸; Zagalskaya & Alexandrov, 2020 ▸). This result confirms the practicability of the *in situ* FTIR cell for electrochemical measurements when electrolyte circulation is rationally used. Based on the LSV results in Fig. 3 ▸, the following suitable voltage parameters for the subsequent *in situ* FTIR test are carefully determined: the OCV (point A; 1.29 versus RHE); the potential at the pre-catalytic stage (point B; 1.40 versus RHE); the onset potential (point C; 1.55 V versus RHE); and the potential at 5 mA cm^−2^ (point D; 1.60 V versus RHE).

**Figure 4:**
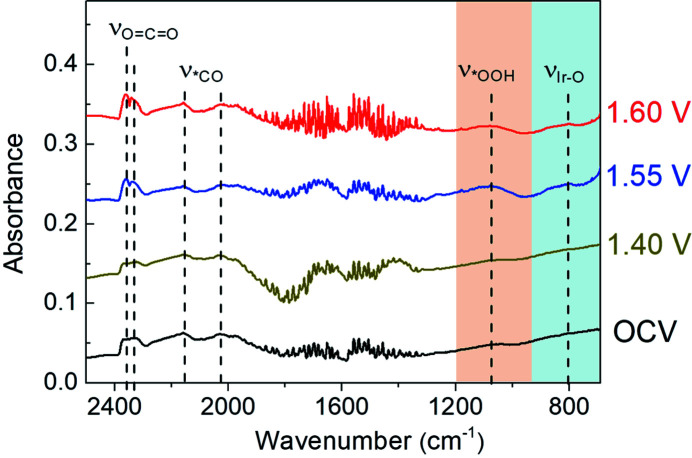
FTIR spectra of the commercial IrO_2_ catalysts during the OER. To clearly present the change of FTIR spectra, a constant *y*-axis offset method is employed to separate plots along the *y*-axis, where the *y*-axis offset values for the plots under OCV, 1.40 V, 1.55 V and 1.60 V are 0, 0.1, 0.2 and 0.3, respectively.

For the FTIR spectra illustrated in Fig. 4 ▸, there are three dominant IR vibrational bands located at ∼2030, 2160 and 2320–2360 cm^−1^ for the IrO_2_ catalysts at OCV, which are well assigned to the stretching modes of bridge-bonded and linear-bonded *CO on the surface of the carbon-based substrate and the stretching model of gaseous CO_2_ in air, respectively (Pate *et al.*, 2022 ▸; Zhang *et al.*, 2021 ▸; Zeng *et al.*, 2022 ▸). When the potential is increased to 1.40 V, no additional vibrational band is obtained for IrO_2_ catalysts relative to that at OCV, indicating that the surface adsorbed species of electrodes at the pre-catalytic stage are similar to those under OCV. Notably, a relatively weak IR vibrational band appears at about 801 cm^−1^ at the enhanced potential of 1.55 V, which may be derived from post-formed Ir—O bonds on the surface of the IrO_2_ catalyst (Su *et al.*, 2021 ▸; Cao *et al.*, 2019 ▸; Jiang *et al.*, 2020 ▸). Interestingly, a strong newly formed IR vibrational band is concurrently observed at about 1079 cm^−1^ at 1.55 V, which is attributed to the formation of a key *OOH intermediate on the surface of the IrO_2_ catalysts (Su *et al.*, 2021 ▸; Zhang *et al.*, 2014 ▸; Czioska *et al.*, 2021 ▸). Moreover, these IR vibrational bands at 801 and 1079 cm^−1^ are retained when the applied potential is further increased to 1.60 V. These results suggest that the *in situ* formed Ir—O bonds on the surface of the IrO_2_ catalyst can effectively trigger the formation of the key *OOH during OER for efficient electrochemical oxygen evolution, where the generation of the key *OOH intermediate may be the rate-determining step for the IrO_2_ catalyst during OER in alkaline solution (Czioska *et al.*, 2021 ▸; Naito *et al.*, 2021 ▸; Jiang *et al.*, 2020 ▸).

**Figure 5:**
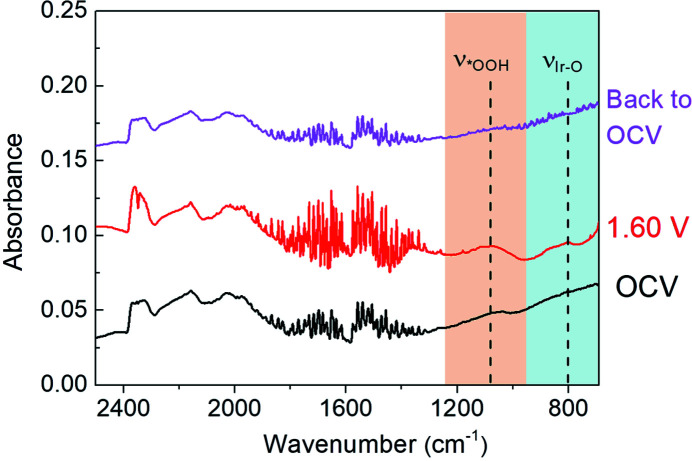
FTIR spectra of the commercial IrO_2_ catalysts under typical potentials. A constant *y*-axis offset method is employed to clearly present the change of the FTIR spectra, with offset values of 0, 0.06 and 0.12 for plots under OCV, 1.60 V and back to OCV, respectively.

To exclude the influence of irreversible structural changes of the catalyst itself, the applied potential is restored to OCV from 1.60 V for the IrO_2_ catalyst (Su *et al.*, 2021 ▸; Ji *et al.*, 2020 ▸). It can be seen in Fig. 5 ▸ that the IR vibrational bands at ∼801 and 1079 cm^−1^, associated with surface Ir—O bonds and the key *OOH formation, have completely disappeared. Moreover, FTIR spectroscopy under the restored OCV shows a similar trend in shape and frequency to that at OCV. This indicates the reversible surface species evolution over the solid–liquid interface of electrodes during OER, and further confirms the formation of the key *OOH intermediate during OER for the IrO_2_ catalyst. Accordingly, it can be revealed that the potential-dependent surface Ir—O bonds may be very helpful for promoting the generation of the key *OOH intermediation during OER, resulting in fast 4 e^−^ OER kinetics for the IrO_2_ catalyst in alkaline solution.

## 5. Conclusion

A novel bifunctional *in situ* FTIR cell has been meticulously designed and prepared, and is compatible with synchrotron-radiation-based IR sources. With well designed IR window and dual electrolyte/gas channels, this *in situ* FTIR cell can be potentially adopted in surface dynamics studies during various electrochemical processes with or without gaseous reactants. By coupling with a single-reflection synchrotron FTIR method, the surface species evolution of commercial benchmark IrO_2_ catalyst is promptly monitored during OER using the *in situ* FTIR cell. The feasibility and practicability of the *in situ* FTIR cell in dynamics studies are rationally demonstrated by the successful observation of the key *OOH intermediate over the IrO_2_ electrode surface during OER. The *in situ* synchrotron FTIR spectroscopic method developed in this work would be very helpful for surface dynamic investigations during important electrocatalytic processes, such as electrocatalytic hydrogen evolution, CO_2_ reduction, N_2_ fixation *etc*.
